# Metabolic Regulators Nampt and Sirt6 Serially Participate in the Macrophage Interferon Antiviral Cascade

**DOI:** 10.3389/fmicb.2019.00355

**Published:** 2019-03-04

**Authors:** Widad Dantoft, Kevin A. Robertson, W. John Watkins, Birgit Strobl, Peter Ghazal

**Affiliations:** ^1^Systems Immunity Research Institute, School of Medicine, Cardiff University, Cardiff, United Kingdom; ^2^Division of Infection and Pathway Medicine, School of Biomedical Sciences, The University of Edinburgh, Edinburgh, United Kingdom; ^3^Institute of Infection and Immunity, School of Medicine, Cardiff University, Cardiff, United Kingdom; ^4^Institute of Animal Breeding and Genetics, Department for Biomedical Sciences, University of Veterinary Medicine Vienna, Vienna, Austria

**Keywords:** cholesterol, metabolism, lipid pathway, sterol, epigenetic, interferon, cytomegalovirus

## Abstract

Molecular determinants underlying interferon (IFN)-macrophage biology can help delineate enzyme systems, pathways and mechanisms for enabling host-directed therapeutic approaches against infection. Notably, while the IFN antiviral response is known to be directly coupled to mevalonate-sterol biosynthesis, mechanistic insight for providing host pathway-therapeutic targets remain incomplete. Here, we show that Nampt and Sirt6 are coordinately regulated upon immune activation of macrophages and contribute to the IFN-sterol antiviral response. *In silico* analysis of the *Nampt* and *Sirt6* promoter regions identified multiple core immune gene-regulatory transcription factor sites, including Stat1, implicating a molecular link to IFN control. Experimentally, we show using a range of genetically IFN-defective macrophages that the expression of *Nampt* is stringently regulated by the Jak/Stat-pathway while *Sirt6* activation is temporally displaced in a partial IFN-dependent manner. We further show that pharmacological inhibition of Nampt and small interfering RNA (siRNA)-mediated inhibition of Nampt and Sirt6 promotes viral growth of cytomegalovirus in both fibroblasts and macrophages. Our results support the notion of pharmacologically exploiting immune regulated enzyme systems of macrophages for use as an adjuvant-based therapy for augmenting host protective pathway responses to infection.

## Introduction

Infection is a dynamically complex and multifaceted process requiring not only the avoidance of immune countermeasures but also the exploitation of host cellular networks and machinery by the pathogen. In many cases, parastization by pathogens and especially by viruses requires remodeling of metabolic and energy resources for the successful production of progeny. Notably, the immune system has been found to cross regulate these resources and processes as an evolutionary selected countermeasure. For example, IFNγ induced consumption of tryptophan, by the Indoleamine 2,3 Dioxygenese (IDO) pathway, has been shown to inhibit replication of several intracellular organism including hCMV (Pfefferkorn, 1984; Carlin et al., 1989; MacKenzie et al., 1998; Bodaghi et al., 1999; Heseler et al., 2013). More recently interferon regulation of the sterol biosynthesis pathway has been shown to be a central biosynthetic pathway targeted by the immune system for broad host-protection against infection.

In this scenario, Toll-like receptor activation of macrophages by pathogens leads to the production of type I interferons which coordinately regulate a marked and sustained reduction in the mevalonate-sterol biosynthetic pathway, and whereby a wide-spectrum of different human and animal viruses have been shown to be sensitive to suppression of the pathway (Adams et al., 2004; Gerbod-Giannone et al., 2006; Eguchi et al., 2008; Zhu et al., 2008; Bauman et al., 2009; Maitra et al., 2009; Haas and Mooradian, 2010; Blanc et al., 2011; Olsen et al., 2011; Chukkapalli et al., 2012; Liu et al., 2013; Mesmin et al., 2013; Reboldi et al., 2014; Singaravelu et al., 2015; Robertson et al., 2016). The currently known molecular pathways for down-regulating the sterol pathway involve the IFN induction of an hydroxylase enzyme (Ch25h) and its cognate regulatory metabolite, 25-hydroxycholesterol (25HC) that potently inhibits, at the protein level, the master transcription factor (TF) for sterol biosynthesis (SREBP2) (Blanc et al., 2013), and also the key regulated mevalonate reductase, HMGCR (Lu et al., 2015), and additionally IFN regulated microRNAs (miR342-5p), that coordinate changes in the enzymatic flux of the cholesterol pathway within the cell (Robertson et al., 2016). However, there remains yet to be identified transcriptional or epigenetic mechanisms for suppression of SREBP2 and sterol biosynthesis.

More broadly, there is increasing evidence showing connections between immune signaling, such as interferon (IFN) signaling, and the regulation of sterol, sugar, and fatty acid metabolism (Zelcer and Tontonoz, 2006; Spann and Glass, 2013; Kotzamanis et al., 2015; York et al., 2015). While the cell typically induces changes through rapid established routes such as the PI3K/AKT/mTOR signaling pathway, these changes are not sustained over a longer period time and do not support the increased needs for *de novo* lipogenesis. In the context of cellular stress and inflammation, Sirtuins (SIRTs) are known to play sustained roles in protecting against cellular stress through epigenetic control of metabolic pathways (Lyssiotis and Cantley, 2012; Jiang et al., 2013). This includes the regulation of glycolytic and lipid metabolism by the nicotineamide adenine dinucleotide (NAD^+^)-dependent deacetylases SIRT1 and SIRT6 (Liu et al., 2012a,b; Elhanati et al., 2013; Tao et al., 2013). Metabolic coupling is strictly dependent on NAD^+^ production through *de novo* biosynthesis from tryptophan or through the nicotineamide (NAM) salvage pathway, which is regulated by the rate-limiting enzyme nicotineamide phosphatidyltransferase (NAMPT). It is notable that NAD^+^-dependent activation of SIRT6 has been shown to repress the *SREBF2* promoter (Elhanati et al., 2013), and thereby directly linking SIRT6 activity to sterol metabolism. However, whether NAMPT or SIRT6 are coordinately regulated by the IFN macrophage antiviral response is not known. Most notably this remains a central unanswered question to the notion of using macrophage interferon biology as a guiderail for identifying host-directed druggable targets as anti-infectives.

In the present report, we find that Nampt and Sirt6 are coordinately regulated upon immune activation of macrophages and contribute to the interferon antiviral response. The coupling to the IFN response is via direct transcriptional activation of NAMPT through the JAK/STAT signaling pathway. We show that pharmacological inhibition of Nampt and small interfering RNA (siRNA)-mediated inhibition of Nampt and Sirt6 enhances the viral growth of cytomegalovirus (mCMV) in both fibroblasts and macrophages. These findings support the proposition that immune regulated enzyme systems may be used as an adjuvant therapy for augmenting the host protective response to infection.

## Results

### Co-ordinate Regulation of Nampt and Sirt6 Are Part of the Interferon-Metabolic Anti-viral Response

**Figure 1:**
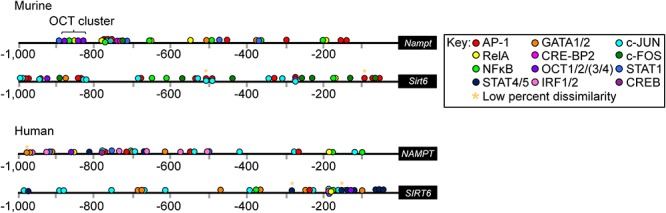
Putative transcription factor (TF) binding sites within the promoter regions of murine *Nampt* and *Sirt6* and human *NAMPT* and *SIRT6*. *In silico* analysis using PROMO of the murine *Nampt* and *Sirt6* and human *NAMPT* and *SIRT6* promoter regions revealed putative binding sites for an array of different immune-regulatory TFs (Supplementary Tables S1–S4). The cut off for dissimilarity to the consensus TF binding-sequence was set to 15% (^∗^ marks binding sites with low percentage, 0–4%, dissimilarity) and the identified string sequences were compared to the consensus binding site sequences for each TF. Figure is not to scale.

We first investgated whether murine *Nampt* and *Sirt6* and human *NAMPT* and *SIRT6* promoter regions contained any putative transcriptional binding sites (TFBS) for immune-regulatory TFs (Figure 1 and Supplementary Tables S1–S4). By using the sequence analysis tool PROMO (Messeguer et al., 2002; Farré et al., 2003), and manual procurement by comparing putative binding sites to published consensus binding sequences, an array of significant binding sites (restricted to 15% dissimilarity) for core immune-activated TFs, including AP-1, NFκB (defined here as DNA binding activity constituted either by p50 homodimer, a p50/p65 heterodimer, or a heterotetramer), RELA (p65 subunit of NFκB), GATA1 and GATA2, were identified within (-1 kb upstream of) the murine *Nampt* and *Sirt6* promoter regions (Figure 1). PROMO analysis of the human *NAMPT* and *SIRT6* promoter regions identified similar binding sites, suggesting that the overall activation mode of these genes is conserved between humans and mice. Notably, several putative Signal Transducers and Activators of Transcription 1 (STAT1) sites were identified across the *Nampt* promoter region, suggesting *Nampt* expression might be driven directly by the activation of the JAK/STAT signaling pathway (Figure 1). A putative Oct cluster [OCT1/2/(3/4)] was also identified in the distal *Nampt* promoter region, in close proximity to putative NFκB, RELA, and STAT1 binding sites. While the promoter region of *Sirt6* did not contain any putative STAT1 binding sites, it was dominated by putative binding sites for Activator protein 1 (AP-1), c-JUN, c-FOS, and NFκB. The AP-1 structure is a heterodimer composed of proteins belonging to the c-FOS, c-JUN, Activating transcription factor (ATF), and Jun dimerization protein (JDP) families (Angel and Karin, 1991; Karin et al., 1997). Consistent with its reported activity, the putative AP-1 binding sites were found in close proximity to either c-FOS, c-JUN, or in areas containing *cis*-located c-FOS and c-JUN binding sites (Figure 1). AP-1, an early response TF, has been reported to regulate gene expression in response to various stimuli, including cytokine stimulation and bacterial and viral infections (Hess et al., 2004).

**Figure 2:**
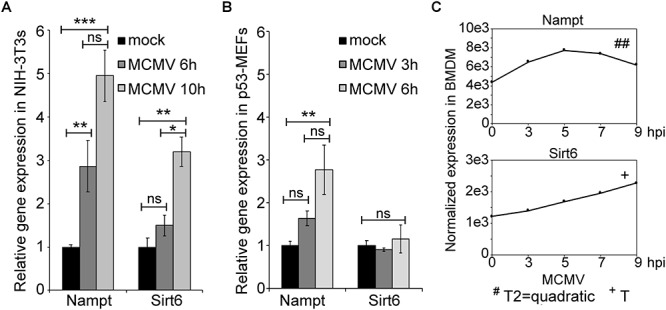
Expression of Nampt and Sirt6 is upregulated by MCMV infection. **(A)** Quantification of relative Nampt and Sirt6 mRNA expression in NIH-3T3 cells, at 6 and 10 h, using RT-qPCR following mCMV infection with mock-infected cells serving as controls (*n* = 3). **(B)** Quantification of relative Nampt and Sirt6 mRNA expression in p53-MEF cells, at 3 and 6 h, using RT-qPCR following mCMV infection (*n* = 3). ANOVA with Tukey post-test was used to assess statistical signficance. **(C)** Normalized temporal *Nampt* and *Sirt6* expression (antilog) in mCMV infected bone marrow derived macrophages (BMDM). The expression was measured over the first 24 h of infection using microarray and compared to timepoint 0. The expression levels between 0 and 10 h were smoothened and fitted to a linear (+), quadatic (#), or cubic (^∗^) polynomal on time and the statistical significance (*p*-values) was assessed. ^+/#/∗^*p* < 0.05, ^++/##/∗∗^*p* < 0.01, and ^+++/###/∗∗∗^*p* < 0.001 were considered to be significant (ns, not significant). Bars represent standard error of the mean (SEM). ^∗^*p* < 0.05, ^∗∗^*p* < 0.01, and ^∗∗∗^*p* < 0.001 were considered to be significant (ns, not significant).

The presence of several putative immune-gene regulatory TF binding sites, prompted us to investigate whether *Nampt* and *Sirt6* expression was induced by infection (Figure 2). The relative expression of *Nampt* and *Sirt6* in mCMV-infected NIH-3T3 and p53 mouse embryonic fibroblasts (p53-MEFs) was measured using Quantitative reverse-transcriptase Polymerase Chain Reaction (qRT-PCR) (Figure 2A,B). mCMV infection of NIH-3T3 and p53-MEFs resulted in significantly higher levels of *Nampt* expression during the first 6 h of infection (Figure 2A,B). While an early increased *Sirt6* expression was not observed, a significantly higher expression was observed in NIH-3T3 after 10 h of infection, indicating a delayed response. Similarly, the temporal expression profiles (over 24 h) of *Nampt* and *Sirt6* were investigated in mCMV infected bone marrow derived macrophages (BMDM). Following mCMV infection, cells were harvested every 2 h until 10 h post-treatment [0 (0 h after viral adsorption or poly(I:C) treatment), 2, 4, 6, 8, 10 h] and at 24 h, followed by transcriptomic profiling and modeling of their temporal expression. In these experiments, polynomial fitting of the smoothened data was used to determine whether the expression profiles of *Nampt* and *Sirt6* changed significantly (where a *R*^2^ > 0.9 indicated significant change) with time in infected BMDM (Supplementary Tables S5, S6). In addition further statistical evaluation was performed by determining the *p*-value of the fitted model in relation to a horizontal flat line, where a significant *p*-value predicted a temporal change and a non-significant *p*-value is predicted of non-fluctuation in expression. Consistent with the observations in NIH-3T3s and p53-MEFs, mCMV infection of BMDM resulted in a significant early and dynamic expression of *Nampt* (Figure 2C and Supplementary Table S5), with a peak at 5 h, followed by a steady decline. Moreover, similarly to the observations in NIH-3T3s and p53-MEFs, temporal expression analysis revealed that mCMV infected BMDM exhibited a delayed but continuous, albeit lower than *Nampt*, significant *Sirt6* expression (Figure 2C and Supplementary Table S6). Collectively, these results show that while *Nampt* and *Sirt6* are both induced in response to mCMV infection, their response time differs from each other irrespective of cell type, indicative of potential differential transcriptional regulation.

### Nampt Gene Expression Is Activated by the Jak/Stat Signaling Pathway and Induced by Both Type-I and Type-II IFNs, While Respone of Sirt6 Is Indirect or Restricted to Type-I IFN Response

**Figure 3:**
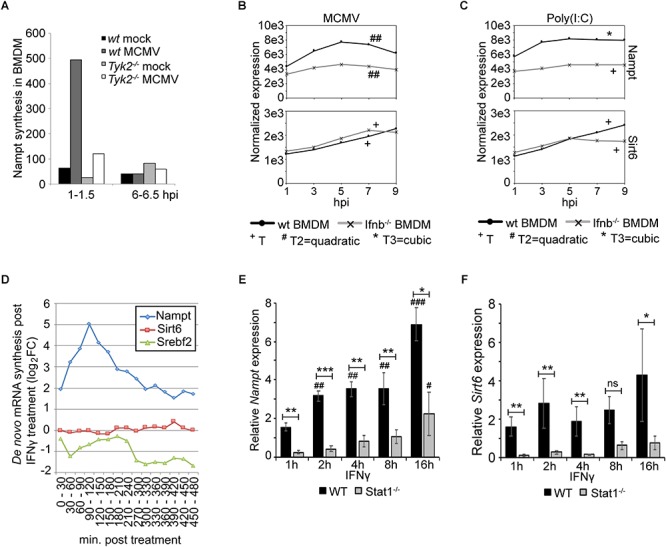
Expression of Nampt is induced by both type-I and type-II IFN, while respone of Sirt6 is restricted to type-I IFN response. **(A)** Quantification of Nampt mRNA expression, using microarray, in wild-type (wt) and *Tyk2^-/-^* BMDM following mock- (control) and mCMV-infection. **(B)** Normalized temporal expression (antilog) of *Nampt* and *Sirt6* in mCMV infected wild-type and *Ifnb^-/-^* BMDM. The expression was measured over the first 24 h of infection using microarray and compared to timepoint 0. The expression levels between timepoints 0–10 h post-streatment were smoothened and fitted to a linear (+), quadatic (#), or cubic (^∗^) polynomal on time to assess significance (*p*-values). ^+/#/∗^*p* < 0.05, ^++/##/∗∗^*p* < 0.01, and ^+++/###/∗∗∗^*p* < 0.001 were considered to be significant (ns, not significant). **(C)** Normalized temporal expression (antilog) of *Nampt* and *Sirt6* in poly(I:C) treated wild-type and *Ifnb^-/-^* BMDM. The expression was measured over the first 24 h using microarray and compared to timepoint 0. The expression levels between timepoints 0–10 h post-streatment were smoothened and fitted to a linear (+), quadatic (#), or cubic (^∗^) polynomal on time to assess significance (*p*-values). ^+/#/∗^*p* < 0.05, ^++/##/∗∗^*p* < 0.01, and ^+++/###/∗∗∗^*p* < 0.001 were considered to be significant (ns, not significant). **(D)** Quantification of *de novo* synthesis of Nampt, Sirt6, and Srebf2 mRNA in IFNγ-stimulated BMDMs using qRT-PCR. Expression was measured every 30 min for a total of 8 h. **(E)** Quantification of relative Nampt mRNA expression in wild-type and *Stat1^-/-^* p53-MEF cells, at 1, 2, 4, 8, and 16 h, using RT-qPCR following IFNγ stimulation (*n* = 3). Expression is relative to wild-type untreated (0 h) cells, set as 1 (not shown). One-way ANOVA with a Tukey’s post-test was used to assess statistical signficance to untreated controls. One-way ANOVA with a Sidak’s multiple comparisons test was used to assess statistical signficance between wild-type and *Stat1^-/-^* mutants. Bars represent SEM. Statisical significance between groups (wild-type and *Stat1^-/-^*) were depicted with ^∗^. Statistical significance in relation to untreated controls (wild-type and *Stat1^-/-^*, respectively) were depicted with #. ^∗^/^#^*p* < 0.05, ^∗∗^/^##^*p* < 0.01, ^∗∗∗^/^###^*p* < 0.001 were considered to be significant. **(F)** Quantification of relative Sirt6 mRNA expression in wild-type and *Stat1*^-^*^/^*^-^ p53-MEF cells, at 1, 2, 4, 8, and 16 h, using RT-qPCR following IFNγ stimulation (*n* = 3). Expression is relative to wild-type untreated (0 h) cells, set as 1 (not shown). One-way ANOVA with a Tukey’s post-test was used to assess statistical signficance to untreated controls. One-way ANOVA with a Sidak’s multiple comparisons test was used to assess statistical signficance between wild-type and *Stat1^-/-^* mutants. Bars represent SEM. Statisical significance between groups (wild-type and *Stat1^-/-^*) were depicted with ^∗^. Statistical significance in relation to untreated controls (wild-type and *Stat1^-/-^*, respectively) were depicted with #. ^∗^/^#^*p* < 0.05, ^∗∗^/^##^*p* < 0.01, ^∗∗∗^/^###^*p* < 0.001 were considered to be significant.

The presence of the several putative STAT1 binding sites in the *Nampt* promoter region suggests that Nampt expression is induced in a JAK/STAT signaling pathway-dependent manner. To initially investigate this, the synthesis of Nampt mRNA was measured *Tyk-2*-deficient BMDM (Figure 3). Nampt mRNA synthesis was investigated in mCMV infected wild-type and Tyk2-deficient BMDM at 1–1.5 h post-infection and at 6–6.5 h post-infection (Figure 3A). The non-receptor tyrosine-protein kinase Tyk2 has been implicated in type-I IFN, IL-6, IL-10, and IL-12 signaling (Velazquez et al., 1992; Stahl et al., 1994; Heinrich et al., 1998; Shimoda et al., 2000; Shaw et al., 2006). Consistent with the identification of putative STAT1 binding sites, mCMV infection of Tyk2-deficient (*Tyk2^-/-^*) BMDM resulted in a much-reduced Nampt synthesis, compared to infected wild-type cells, suggesting that *Nampt* is, at least partly, induced in a JAK/STAT pathway-dependent manner (Figure 3A).

The presence of putative STAT1 binding sites and the observed dependence of *Nampt* expression on TYK2 and on the JAK/STAT signaling pathway poses the question *Whether the induced expression of Nampt and Sirt6 is dependent on type I IFN signaling?* To investigate this, the expression of *Nampt* and *Sirt6* was assessed in polyinosinic:polycytidylic acid [poly(I:C)] treated *Ifnb*-deficient BMDM and compared to the response in mCMV infected *Ifnb1*-deficient (C57BL/6J *Ifnb1^-/-^*) BMDM (Figure 3B,C). Poly(I:C), a ligand of Toll like receptor 3 (TLR3), is structurally similar to double-stranded RNA and is, thus, used to simulate viral infections. Following mCMV infection or poly(I:C) treatment, cells were, as decribed above (Figure 2), harvested every 2 h until 10 h post-treatment and at 24 h, followed by transcriptomic profiling and modeling of their temporal expression. As in Figure 2C, mCMV infection of wild-type BMDM resulted in an early dynamic expression of Nampt. Similar to *Nampt*, mCMV infection of wild-type BMDM significantly induced, albeit at a lower level, the expression of *Sirt6* expression peaking downstream of Nampt. Poly(I:C) treatment of wild-type BMDM, resulted in a significant temporal activation of both *Nampt* and *Sirt6*, indicating that the observed expression is a host-driven response to infection (Figure 3C and Supplementary Tables S7, S8). The level of *Nampt* activation following mCMV infection was significantly reduced in *Ifnb1*-deficient cells, suggesting that a robust *Nampt* expression response is IFNβ-dependent (Figure 3B). While the robustness in *Nampt* expression was lost, a small significant temporal change in the expression profile was observed, suggesting that *Nampt* expression is possibly governed by other factors or pathways including by other type I Ifns but that the magnitude of expression is strongly dependent on intact IFNβ-signaling. The early expression of Sirt6 was, however, not extensively altered in mCMV infected or poly(I:C) treated *Ifnb*-deficient cells. The expression was similar to wild-type cells up until 7 h post-infection and up until 5 h post-poly(I:C) treatment. This was followed by a reduction in expression, indicating that early but not late activation of *Sirt6* expression following mCMV infection and poly(I:C) treatment is induced independently of IFNβ (Figure 3B,C and Supplementary Tables S6, S8). Together, these results indicate that Nampt and partially Sirt6 are coupled to the type I IFN response in macrophages.

Further in our BMDM, where stimulation with physiologically relevant concentration of IFNγ has been previously determined (Kropp et al., 2011), IFNγ induced the expression of *Nampt*, peaking at 6 h post-treatment (Supplementary Figure S1 and Table S9). Moreover, to further investigate the dependence of *Nampt* and *Sirt6* expression on the type-II IFN response, the level of newly transcribed *Nampt* and *Sirt6* mRNA was measured every 30 min, over a period of 8 h, using reverse transcriptase-quantitative PCR (qRT-PCR) in BMDM stimulated with IFNγ (Figure 3D). Stimulation with IFNγ resulted, after 2 h of infection, in an eight-times increase in *de novo* transcribed *Nampt* RNA levels, followed by a rapid drop in *Nampt* mRNA. *Sirt6* mRNA levels were on the other hand not affected by IFNγ stimulation, further suggesting that it is not a type-II IFN stimulated gene. Notably, the increase in *Nampt* mRNA expression was followed by a drop in *Srebf2* expression, consistent with previously published data from Blanc et al. (2011).

The dependence on type-II IFN and JAK/STAT signaling was further investigated in wild-type and Stat1-deficient (*Stat1^-/-^*) p53-MEFs stimulated with IFNγ (Figure 3E,F). Steady state levels of *Nampt* mRNA was investigated at 1, 2, 4, 8, and 16 h post-treatment and compared to untreated (0 h) cells (Figure 3E). IFNγ activated of wild-type cells resulted, as early as 2 h post-treatment, in a significantly increased expression of *Nampt* compared to untreated cells (statistical significance depicted with #), suggesting that *Nampt* is a type-I IFN responsive gene. In the IFNγ activated Stat1-deficient (*Stat1^-/-^*) cells, a significantly increased *Nampt* expression, compared to the untreated Stat1-deficient control (not shown), was only observed at later time points (16 h). This expression was significantly reduced at all time points compared to activated wild-type cells, indicating that *Nampt* is dependent on intact Stat1 signaling, consistent with the identification of putative STAT1 binding sites within the *Nampt* promoter region.

Moreover, loss of Stat1 resulted in a significantly reduced expression of Sirt6 compared to the wild-type p53-MEFs (Figure 3F). Notably, unlike *Nampt*, Sirt6 mRNA expression in wild-type cells did not increase statistically with time (compared to untreated control), suggesting that it is not a type-II IFN stimulated gene. Nor did the expression change significantly in Stat1-deficient cells to the respective untreated control. It is possible to speculate that Stat1 signaling is required for the basal, but not induced, *Sirt6* expression. The absence of identified STAT1 binding sites in combination with the absence of gene induction with time further support the notion that this activation is indirect. Collectively, these results show that *Nampt* and *Sirt6* are both induced in response to mCMV infection and suggest that *Nampt* is an interferon-stimulated gene (ISG), with *Nampt* expression being an immediate-response gene induced by type-I and type-II IFN in a JAK/STAT dependent manner.

### Intact Type-I IFN Signaling Is Required for Strong Infection-Induced Expression of Upstream, but Not Downstream, TLR Signaling Pathway Components

**Figure 4:**
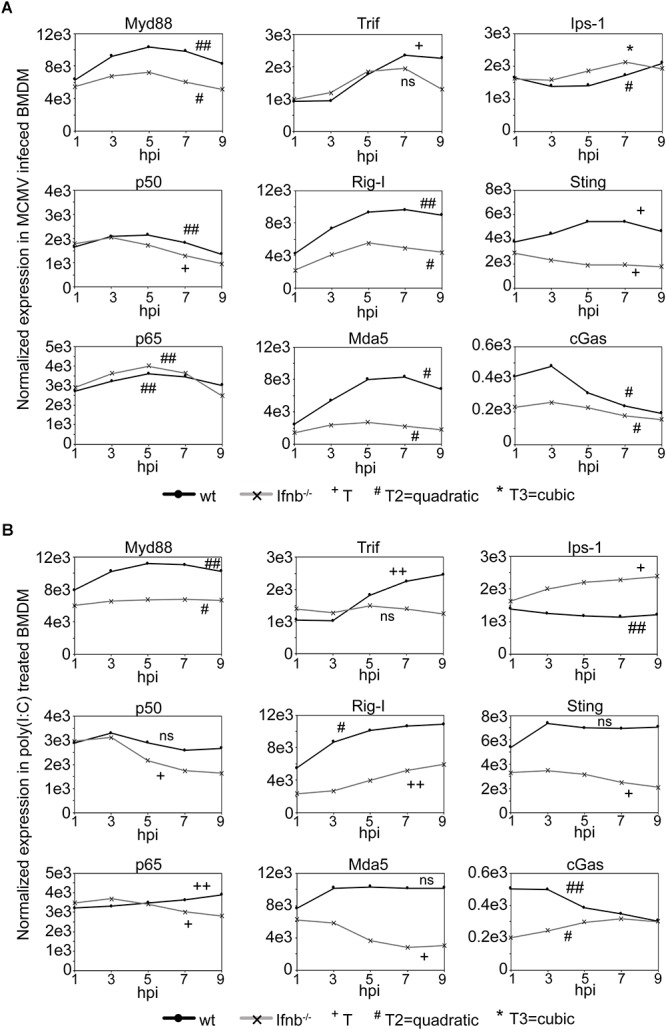
Expression of upstream, but not downstream, TLR signaling pathway components is dependent on IFNβ/type-I IFN signaling. **(A)** Normalized temporal expression (antilog) of *Myd88*, *p50* (*Nfkb1*), *p65* (*Rela*), *Trif* (*Ticam1*), *Rig-I* (*Ddx58*), *Mda-5* (*Ifih1*), *Ips-1* (*Mavs*), *Sting* (*Tmem173*), and *cGas* (*Mb21d1*) in mCMV infected wild-type and *Ifnb*^-^*^/^*^-^ BMDM. The expression was measured over the first 24 h of infection using microarray and compared to timepoint 0. The expression levels between timepoints 0–10 h post-streatment were smoothened and fitted to a linear (+), quadatic (#), or cubic (^∗^) polynomal on time to assess significance (*p*-values). ^+/#/∗^*p* < 0.05, ^++/##/∗∗^*p* < 0.01, and ^+++/###/∗∗∗^*p* < 0.001 were considered to be significant (ns, not significant). **(B)** Normalized temporal expression (antilog) of *Myd88*, *p50* (*Nfkb1*), *p65* (*Rela*), *Trif* (*Ticam1*), *Rig-I* (*Ddx58*), *Mda-5* (*Ifih1*), *Ips-1* (*Mavs*), *Sting* (*Tmem173*), and *cGas* (*Mb21d1*) in poly(I:C) treated wild-type and *Ifnb*^-^*^/^*^-^ BMDM. The expression was measured, as in **(A)**, over the first 24 h using microarray and compared to timepoint 0. The expression levels between timepoints 0–10 h post-streatment were smoothened and fitted to a linear (+), quadatic (#), or cubic (^∗^) polynomal on time to assess significance (*p*-values). ^+/#/∗^*p* < 0.05, ^++/##/∗∗^*p* < 0.01, and ^+++/###/∗∗∗^*p* < 0.001 were considered to be significant (ns, not significant).

Infection with double-stranded DNA viruses, such as CMV, are known to trigger the common TLR signaling pathway that elicits the activation of NFκB and MAPK through the Myd88 adaptor (Compton et al., 2003), while other pathways, such as the IPS-1 and STING mediated pathways, induce type-I IFN synthesis (Seth et al., 2005; Cheng et al., 2007; Ishikawa et al., 2009) resulting in downstream target activation. To explore the gene activation of factors belonging to these pathways, the temporal gene expression profiles of *Myd88*, *p50* (*Nfκb1*), *p65* (*Rela*), *Trif* (*Ticam1*), *Rig-I* (*Ddx58*), *Mda5* (*Ifih1*), *Ips-1* (*Mavs*), *Sting* (*Tmem173*), and *cGas* (*Md21d1*) were investigated in mCMV infected or poly(I:C) treated wild-type and *Ifnb1*-deficient BMDM (Figure 4 and Supplementary Tables S10–S27). Following mCMV infection of wild-type BMDM, a significant temporal activation of *Myd88*, *p50*, *p65*, *Trif*, *Rig-I*, *Mda5*, and *Sting* was observed (Figure 4A). The expression profile of *Ips-1* was initially suppressed up until 5 h post-infection followed by an activation, while *cGas* exhibited an early activation between 1 and 3 h post-infection, followed by a rapid drop in expression. Notably, the activation of *Trif*, which was absent until 3 h post-infection, was followed by a rapid increase in expression peaking at 7 h. In *Ifnb1*-deficient BMDM, mCMV infection resulted in a significant temporal change in expression of all genes but *Trif*. While the temporal expression of *Myd88*, *Rig-I*, *Mda-5*, and *Sting* was significantly changed over time, the level of expression was much reduced in these cells, suggesting that IFNβ-signaling is in part needed for the full induction of these genes. Notably, while the expression level of *cGas* was initially much higher in wild-type cells, the level expression after 5 h dropped to similar levels as those observed in the *Ifnb1*-deficient cells, suggesting that IFNβ is required for the early activation of this gene.

In poly(I:C) activated wild-type BMDM, significant temporal expression change was observed for *Myd88*, *p65*, *Trif*, *Rig-I*, *Ips-1*, and *cGas*, consistent with that observed in mCMV infected cells (Figure 4B). *p50*, *Mda5*, and *Sting* all exhibited an initial increase in expression between 1 and 3 h post-treatment, however, unlike *Mda5* and *Sting* that did not significantly change, the level of *p50* expression was reduced between 3 and 7 h. The modeled temporal change in the *p50* expression profile was, however, not significant. In *Ifnb1*-deficient BMDM, a significant temporal change was observed for all genes with the exception of *Trif*. As in infected *Ifnb1*-deficient BMDM, poly(I:C) treatment resulted in a reduced temporal expression for *Myd88*, *Rig-I*, *Mda-5*, and *Sting*. Moreover, *Mda5* exhibited a repressed temporal profile, as compared to its expression in poly(I:C) treated wild-type cells. Notably, *Ips-1* and *cGas* both exhibited an increased expression over time, with the expression of *Ips-1* exceeding that observed in wild-type cells (Figure 4B).

Collectively, these results suggest intact IFNβ-signaling is not required for the expression of *p50* and *p65* following mCMV infection, but is required for the magnitude in expression of the upstream components (*Myd88*, *Rig-I*, *Mda5*, and *Sting*) of these pathways.

### Inhibition of SIRT6 and NAMPT Results in Increased Viral Replication

**Figure 5:**
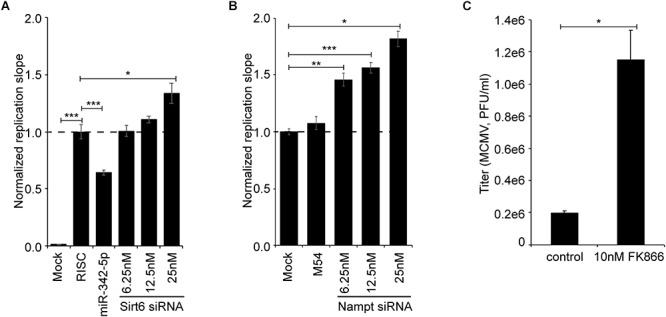
Inhibition of NAMPT and SIRT6 activity increases viral loads in MCMV infected cells. **(A)** Normalized replication slope of mCMV-GFP in Sirt6 siRNA-treated NIH-3T3. “RISC-free” siRNA served as negative control and miR342-5p siRNA served as positive control. **(B)** Normalized replication slope of mCMV-GFP in Nampt siRNA-treated NIH-3T3, with M54 serving as positive control. Bars represent SEM. Statistical significance was determined using One-way ANOVA with a Dunnett’s multiple comparisons test. ^∗^*p* < 0.05, ^∗∗^*p* < 0.01, and ^∗∗∗^*p* < 0.001 were considered to be significant (ns, not significant). **(C)** mCMV-GFP replication (titer) in NIH-3T3 after pharmacological inhibition of NAMPT with FK866 (10 nM final concentration). mCMV-GFP was propagated in NIH-3T3 fibroblasts and quantified by plaque assay on p53-MEF monolayers in 48-well plates. Statistical significance was determined using unpaired *t*-test with Welch’s correction. ^∗^*p* < 0.05 was considered to be significant.

The observation that Nampt and Sirt6 were coordinately induced in macrophages by immune stimulation, either by infection or the ensuing interferon response, prompted us to test whether NAMPT and SIRT6 exhibit antiviral activity. To investigate whether Sirt6 and Nampt exhibit antiviral properties, mCMV replication was measured after siRNA mediated knockdown of Sirt6 and after pharmacologic inhibition or siRNA mediated knockdown of Nampt, with the highly specific non-competitive inhibitor FK866, respectively (Hasmann and Schemainda, 2003) (Figure 5 and Supplementary Figure S2). Consistent with the reported antiviral activity of human SIRT6 (Koyuncu et al., 2014), mediated knockdown of murine Sirt6 resulted, in a siRNA concentration-dependent manner, in an increased viral replication (Figure 5A). Moreover, siRNA mediated knockdown and pharmacologic inhibition of murine Nampt also resulted in an increase in viral replication, respectively (Figure 5B,C). Together, these results indicate that Sirt6 and Nampt both display antiviral properties, providing druggable targets in bolstering interferon antiviral immunity linked to sterol metabolism.

## Discussion

**Figure 6:**
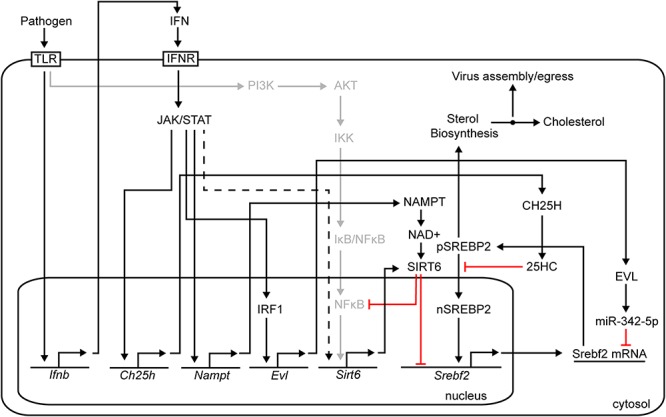
Summary figure.

Here, we demonstrate upon infection of macrophages the serial activation of *Nampt* and *Sirt6*. The observed rapid kinetics of *Nampt* induction shows a strict dependency on both type I and type II IFN signal activation of transcription and, thus, represents an immediate-early class of ISG. By contrast *Sirt6* shows delayed induction kinetics and is only indirectly activated downstream of viral induced type I IFN signaling. In agreement, we find the *Nampt* promoter region contains multiple consensus Stat1 binding sites whereas these sites are absent in the *Sirt6* promoter region. Notably, pharmacological inhibition of NAMPT enzymatic activity or knock-down of Nampt or Sirt6 result in increased viral replication revealing anti-viral roles for these metabolic regulators in infection. Hence, in an apparent orchestrated and coordinated manner Nampt enzymatically drives NAD^+^ production that is a key rate-limiting co-factor for Sirt6 activation and thereby couples Sirt6 functions to the IFN antiviral response (Figure 6).

We further find that temporal expression analysis of key pathway components of the common TLR signaling pathway, which elicits the activation of NFκB and MAPK through the Myd88 adaptor (Compton et al., 2003), and the IPS-1 and STING mediated pathways that induce type-I IFN synthesis, revealed a part dependency on intact type I IFN signaling as loss of *Ifnb1* resulted in a reduced magnitude of expression (*Myd88*, *Rig-I*, *Mda5*, and *Sting*). The downstream signaling components of these pathways, *p50*, *p65*, *Trif*, *Ips-1*, and *cGas* were on the other hand not affected in the same way by the loss of *Ifnb1*. An activated expression profile was, however, observed for *Ips-1* and *cGas*, suggesting that intact type I IFN signaling might be required for maintaining a regulated expression of these genes. As for *p50* (*NFκB*), an initial increased expression was observed followed by a gradual (mCMV infection) or rapid (poly(I:C) treatment) declining expression. A similar expression profile was observed for *p65* following mCMV infection, but not poly(I:C) treatment. Notably, in recent years, SIRT6 has been shown to inhibit NFκB expression (Kawahara et al., 2009; Santos-Barriopedro and Vaquero, 2018; Santos-Barriopedro et al., 2018) and NFκB target gene activation by interacting with p65 (Figure 6) (Li et al., 2018; Santos-Barriopedro and Vaquero, 2018). It is possible that this mode of regulation is reflected in the observed *p50* and *p65* expression profiles, nevertheless, further analysis would be required to confirm this. Whether *Sirt6* is regulated by NFκB in this system remains to be explored, however, global profiling of p65 binding sites (by ChIP-seq) in TNFα-induced human osteosarcoma U-2 OS cells (Janus et al., 2018) and TNFα-induced or poly(I:C) stimulated Detroit 562 cells (Borghini et al., 2018) did not identify *SIRT6* as a NFκB/p65 target gene. Genome-wide profiling of p65-bound sites after 3 and 6 h of LPS treatment, have on the other hand identified *NAMPT* as a p65-activated gene (Lim et al., 2007).

Together, these findings are consistent with observations, in other systems, that are supportive of a potential antiviral role for Nampt and Sirt6 (Schoggins et al., 2011; Van den Bergh et al., 2012; Chen et al., 2013; Koyuncu et al., 2014; Zhang et al., 2014; Li et al., 2018). In a systems-level screen for ISGs with antiviral activity, human NAMPT was identified as one of several type-I ISGs that exhibited, in infected Huh-7 cells, antiviral activity toward Venezuelan equine encephalitis virus (VEEV), a single-stranded RNA virus (Schoggins et al., 2011). NAMPT has also been reported to exhibit anti-HIV-1 activity, interfering with both early events of the life cycle (Van den Bergh et al., 2010) and Tat-induced HIV-1 long terminal repeat (LTR) transactivation (Zhang et al., 2010; Chen et al., 2013; Zhang et al., 2014). As for the role of SIRT6 in antiviral immunity this is less known. Koyuncu et al. (2014) reported in a loss-of-function study, that loss of human Sirtuin activity, including SIRT6 activity, in infected fibroblast MRC5 cells resulted, by unknown mechanism, in significant increases in viral titers (hCMV, HSV-1, Adenovirus, and Influenza A). Moreover, a recent report by Li et al. (2018) show that SIRT6 negatively regulates Dengue virus-induced inflammatory responses by targeting the DNA binding domain of NFκB p65. Notably, unlike our observations reported here those reported by Koyuncu et al. (2014), DENV replication was reduced in HEK293T cells upon silencing of SIRT6 (Li et al., 2018). It is possible that with the diverse nature of SIRT6 the mechanisms by which it exerts its antiviral function differs depending on cell type and viral strain, though the mechanism by which Nampt and Sirt6 exert antiviral effects in these studies is not known. Nevertheless, together with our findings they support the notion that NAMPT and SIRT6 constitute yet another way by which the macrophage can limit productive viral infection.

A central mechanism of action worth noting is the reciprocal increase in *de novo* Nampt mRNA expression, in IFNγ stimulated BMDM, is followed by a decrease in *Srebf2* transcription. This is consistent with studies demonstrating IFN-antiviral suppression of transcription of multiple members of the sterol biosynthesis pathway, in part mediated by a drop in SREBP2 RNA transcription and protein levels (Blanc et al., 2011). Mechanistic studies of IFN suppression of macrophage sterol biosynthesis pathway have determined an approximately 40% contribution by Ch25h and its cognate metabolite 25HC acting at the post-translational level, and 40% by a post-transcriptional mechanism involving microRNA (miR342-5p). However, these known mechanisms fail to account for the observed transcriptional effects on Srebf2 levels. In this regard, there is good evidence to show that Sirt6 binds to and regulates Srebf2 via transcription (Lu et al., 2015; Robertson et al., 2016). Figure 6 shows a schematic of a proposed mechanism for the anti-viral activies of Nampt and Sirt6, mediated through epigentic transcriptional suppression of *Srebf2*, which encodes the master TF sterol biosynthesis. Figure 6 also highlights the other proposed molecular pathways for down-regulating the sterol pathway in macrophages involving the generation of 25-hydroxycholesterol (25HC) and miR342-5p microRNA, both of which contribute toward modulating the SREBP2 autoregulatory loop in response to interferon-signaling (Reboldi et al., 2014; Robertson et al., 2016).

It is noteworthy that host-directed targeting of immune modulated cellular pathways can be used as an innovative therapeutic intervention that also overcomes the antiviral drug resistance (Ghazal et al., 2000). In this regard, we note that SIRT6 inhibitors are under development as anti-cancer drugs (Hu et al., 2014; Parenti et al., 2014; Sociali et al., 2015). Some studies have investigated the efficiency of Ex527 (Selisistat), a commercially available Sirtuin inhibitor (Sigma-Aldrich) (Lugrin et al., 2013; Ekblad and Schüler, 2016) and another proposed approach in inhibiting SIRT6 activity is through administration of nicotineamide (NAM/Vitamin B3), which in addition to being a NAD^+^ precursor, also acts as an endogenous, non-competitive Sirtuin inhibitor (Bitterman et al., 2002). Thus, there is an opportunity for repurposing these cancer drugs for potential antiviral therapy.

## Materials and Methods

### Mice

C57BL/6 mice were housed in the specific pathogen-free animal facility at the University of Edinburgh. Tyk2^-/-^ mice were maintained under specific-pathogen-free conditions at the Institute of Animal Breeding and Genetics, Department for Biomedical Sciences, University of Veterinary Medicine Vienna, Vienna, Austria. The generation or source of knockout mouse strains for Tyk2^-/-^ has been described before (Strobl et al., 2005). All procedures were carried out under project and personal licenses approved by the Secretary of State for the Home Office, under the United Kingdom’s 1986 Animals (Scientific Procedures) Act and the Local Ethical Review Committee at Edinburgh University.

### Cell Propagation and Culture

NIH-3T3 (ATCC^®^ CRL-1658^TM^) immortalized cell line of embryonic mouse fibroblasts was obtained from American Type Culture Collection (ATCC) (Manassas, VA, United States) and grown in Dulbecco’s modified Eagle medium (DMEM) (Lonza, Verviers, Belgium), supplemented with 5% Calf Serum (CS) (Thermo Fisher Scientific, Waltham, MA, United States), 2 mM glutamine (Lonza) and 50 U/ml of penicillin/streptomycin (Lonza). The p53-MEF immortalized cell lines, of p53^-/-^ embryonic mouse fibroblasts [p53-MEFs, MB355 (ATCC^®^ CRT-2818^TM^)] was obtained from American Type Culture Collection (ATCC) (Manassas, VA, United States). p53-MEFs and *Stat1*^-/-^ p53-MEFs were grown in DMEM (Lonza), supplemented with 5% fetal calf serum (FCS) (Thermo Fisher Scientific), 2 mM glutamine (Lonza) and 100 U/ml of penicillin/streptomycin (Lonza). BMDM were isolated and grown in DMEM/F-12 (Ham 1:1) and L-glutaMAX, supplemented with 10% Fetal Calf Serum (Lonza), 10% L929 and 100 U/ml of penicillin/streptomycin. All cells were grown in accordance to standard procedures. BMDMs were differentiated with CSF-1 derived from L929 cells for 7 days prior to further treatment.

### Viruses and Reporter Viruses

Wild-type murine cytomegalovirus (MCMV-C3X) has been previously described (Messerle et al., 2000). The GFP-encoding MCMV (mCMV-GFP) has also been previously described (Angulo et al., 2000). For RNA expression analysis, infection was done at a multiplicity of index (MOI) of 1 unless else specified.

### RNAi and Assay for GFP-Virus Growth

Small interfering RNA and “RISC-free” control siRNA were purchased from Dharmacon^®^ RNAi Technologies (Thermo Fisher Scientific). miR-342-5p microRNA mimic were kindly gifted by Integrated DNA Technologies (WOS:000332467100005). The following siRNAs were used: “RISC-free” siRNA, SiGenome^TM^ Control (Cat. No. D-001220-01-05); Mouse Sirt6 siRNA (deconvoluted), ON-TARGETplus siRNA Mouse Sirt6 (Cat. No. J-061392-09, J-061392-10, J-061392-11, J-061392-12); Mouse Nampt siRNA (deconvoluted), ON-TARGETplus siRNA Mouse Nampt (Cat. No. J-040272-09, J-040272-10, J-040272-11, J-040272-12); M54 siRNA, custom made order from Dharmacon^®^ (5′–3′ sense strand sequence is AGAAAGACGACCTGAGCTA). Mimics and siRNA were transfected into cells (NIH-3T3), in a 96 well plate, using DharmaFECT1 (Thermo Fisher Scientific) using the reverse-transfection method and in accordance to the manufacturer’s recommendations. M54 siRNA and miR-342-5p microRNA mimic were transfected at a final concentration of 25 nM and Sirt6 siRNA was transfected at a final concentration of 6.25, 12.5, and 25 nM/well. For the analysis of miR-342-5p inhibitor effects on virus replication, medium containing 3% delipidized serum [Bovine Serum, Lipid Depleted (Part number: S181L), VWR, United Kingdom] was used. After 48 h, MCMV-GFP (MOI 0.025) was used for infection. The viral growth (fluorescence in each well) was measured using a POLARstar OPTIMA plate reader (BMG Labtech, Aylesbury, United Kingdom) according to manufacturer’s recommendations. The RNAi and viral growth assay were set up as two independent experiments with 3 biological replicates per experiment (*n* = 6). Virus replication slopes over the linear phase were calculated, from 68 h to the end of the time course, and then normalized to control transfected wells. Statistical significance was determined using One-way ANOVA with a Dunnett’s multiple comparisons test. *p*-Values of <0.05, <0.01, and <0.001 were considered significant.

### *In silico* Promoter Analysis

*In silico* promoter analysis of the NAMPT and SIRT6 promoter regions was done using PROMO, a virtual laboratory for identification of putative transcriptional binding sites (Messeguer et al., 2002; Farré et al., 2003). The promoter regions consisting of the 1 kb upstream regions of murine and human NAMPT and SIRT6 were retrieved from the Mouse Genome Informatics (MGI) Web Site (Bult et al., 2015; Finger et al., 2017; Smith et al., 2018) and The National Center for Biotechnology Information (NCBI) (Geer et al., 2010) resource, and imported into the online PROMO analysis tool. Species specific (*Mus musculus* or *Homo sapiens*) TFs and TF sites were chosen. For murine *Nampt* and *Sirt6*, 306, and 270 putative TF binding sites within a dissimilarity margin less or equal than 15% were identified in the promoter regions, respectively. For human *NAMPT* and *SIRT6*, 444 and 436 putative TF binding sites within a dissimilarity margin less or equal than 15% were identified in the promoter regions, respectively. All identified putative binding sites can be found in Supplementary Tables S1–S4. From these, the most probably immune-regulatory and core TF binding sites were identified via manual procurement by comparing putative binding site to publicly available/published consensus binding sequences for each TF.

### IFN-γ Treatment of p53-MEFs and Isolation

Wild-type and *Stat1*^-^*^/^*^-^ p53-MEFs were plated, in a 24-well plate, at a cell density of 3 × 10^5^ cells/well and grown in DMEM (Lonza), supplemented with 5% FCS (Thermo Fisher Scientific), 2 mM glutamine (Lonza) and 100 U/ml of penicillin/streptomycin (Lonza), for 24 h prior to treatment with murine recombinant IFN gamma (IFNγ) and (Perbio Science). The IFNγ was diluted in complete medium and added to cells at a final concentration of 10 U/ml. Cells were harvested at 1, 2, 4, 8, and 16 h post-treatment for quantitative real-time PCR analysis using 350 μl Qiagen RTL Plus buffer (Qiagen RNeasy Plus kit) as per manufacturer’s recommendations.

### BMDM IFN-γ Treatment, RNA Labeling, and Isolation

Incorporation of 4-thiouridine (Sigma-Aldrich, St. Louis, MI, United States) into newly-transcribed RNA was undertaken as described by Dölken et al. (2008) and Robertson et al. (2016). In brief, at time zero medium was aspirated from all plates and 15 ml of pre-warmed medium containing IFN-γ (final concentration of 10 U/ml) or normal medium was added to the cultures. RNA labeling in BMDM during the first 30 min of the time course was undertaken by addition of 200 μM 4-Thiouridine to the medium of appropriate plates at this time. After 30 min, to end the RNA labeling period, terminate transcription and lyse the cells, medium was aspirated from the labeled BMDM and replaced with 4 ml of RLT lysis buffer (Qiagen, Hilden, DE, United States). In parallel, 10 ml of medium from the next BMDM cultures to be labeled was added to an appropriate volume of 4-thiouridine, mixed and immediately added back to the plate. BMDM cultures were then returned to the incubator. The above cycle of 4-thiouridine addition to BMDM cultures and transcriptional termination was repeated at 30-min intervals until the end of the time course. Total RNA was isolated using RNeasy Midi kit (Qiagen) according to manufacturer’s instructions, quantitated using a Nanodrop (Thermo Scientific) and integrity was confirmed using an Agilent Bioanalyser (Agilent United Kingdom). Newly transcribed RNA (ntRNA) was then isolated as described in Dölken et al. (2008) and Robertson et al. (2016) and again quantitated using a Nanodrop, followed by qRT-PCR.

### Quantitative Real-Time PCR (qRT-PCR) Analyses of Individual Genes

Cells were harvested in 350 μl Qiagen RTL Plus buffer (Qiagen RNeasy Plus kit) as per manufacturer’s recommendations. Total RNA was extracted from cells with RNeasy Plus kit (Qiagen) according to the manufacturer’s instructions and quantitated using a Nanodrop (Thermo Scientific). All experiments were performed on three biological replicates/samples (*n* = 3) and expression analysis were performed with three technical replicates/sample (*n* = 3), unless else specified. Quantitative gene-expression analyses were performed using Taqman^®^ Primer probe sets (Applied Biosystems, Warrington, United Kingdom). Mouse Assay ID: NAMPT: Mm00451938_m1, SIRT6: Mm01149042_m1, SREBF2: Hs01081784_m1, Actin Beta (ACTB FAM): Mm02619580_g1. Quantitative real-time PCR was performed either as one-step reactions (qRT-PCR) or two-step reactions (RT-qPCR) with an initial separate cDNA synthesis step. qRT-PCR and RT-qPCR were performed in a Stratagene MX3000P machine (Stratagene California, San Diego, CA, United States). For qRT-PCR, each sample reaction was performed in 10 μl volumes using 96-well Non-Skirted, White PCR Plates (ABgene, United Kingdom) and MicroAmp Optical Caps (Applied Biosystems, United Kingdom). For one reaction, 50 ng of diluted total RNA samples was added to 2.5 μl of 4x qScript One-Step Fast qRT-PCR (Low ROX) master-mix, 0.5 μl qScript One-step Reverse Transcriptase (Cat. No. 95081, Quanta Biosciences, United States), 0.5 μl of Taqman primer/probe set (Applied Biosystems), and RNase-free H_2_O to a total volume of 10 μl. cDNA synthesis by reverse-transcription was performed at 50°C for 5 min, followed by initial denaturation at 95°C for 30 s, and 40 cycles of combined denaturation at 95°C for 3 s followed by annealing/primer extension (detection) at 60°C for 30 s. For two-step analysis, 500 ng of isolated total RNA was used for cDNA synthesis with random hexamers using SuperScript^®^ III Reverse Transcriptase (Thermo Fisher Scientific) following the manufacturer’s instructions. Following cDNA synthesis, qPCR was performed in 10 μl volumes. For one reaction, 50 ng cDNA (equivalent to 50 ng total RNA) was added to 5 μl of 2x PerfeCta^®^ qPCR ToughMix^TM^ (Low ROX) master-mix, 0.5 μl of Taqman primer/probe set (Applied Biosystems), and RNase-free H_2_O to a total volume of 10 μl. Expression of target genes was normalized to ActB. The normalized data were used to quantify the relative levels of a given mRNA according to comparative cycle threshold (2^-ΔΔ^CT) analysis (Livak and Schmittgen, 2001; Schmittgen and Livak, 2008). Statistical significance in Nampt and Sirt6 expression was calculated using One-way ANOVA with a Tukey’s or Sidak’s multiple comparisons test (between wild-type and *Stat1*^-^*^/^*^-^ p53-MEFs). Statistical significance for knock-down efficiency of Nampt and Sirt6 was determined using One-way ANOVA with a Dunnett’s multiple comparisons test. *p*-Values of < 0.05, < 0.01, and < 0.001 were considered significant.

### Pharmacological Inhibition of NAMPT With FK866 and Viral Plaque Assay

Pharmacological inhibition of NAMPT was done using FK866 (Hasmann and Schemainda, 2003) at a final concentration of 10 mM. Briefly, NIH-3T3 cells were seeded in a 48 well plate at a cell density of 1 × 10^5^ cells/well and infected with MCMV-GFP (MOI 0.1) the following day. After the adsorption period, the infection media was replaced with media with FK866 (10 nM) or without (control). Cells were assessed for viral growth (GFP signal) over a 4-day period at which point the cells were harvested and frozen down at -80°C. The effect of FK866 treatment on viral growth was quantified by plaque assay on p53^-/-^ MEF monolayers in 48-well plates using standard methodology. Statistical significance was determined using unpaired *t*-test with Welch’s correction. *p*-Values of < 0.05, < 0.01 and < 0.001 were considered significant.

### Time-Course Expression Analysis

Temporal expression analysis for Nampt, Sirt6, and Stat1 in wild-type and IFNB^-/-^ BMDM are based off previously generated microarray data published by Blanc et al. (2013). Briefly, wild-type, and IFNB^-/-^ BMDM were either infected with MCMV (MOI 1) or treated with or poly(I:C) (25 μg/ml) or IFNγ (10 U/ml). Cells infected with MCMV or treated with poly(I:C) were harvested at 0, 2, 4, 6, 8, 10, and 24 h post-treatment for RNA isolation and microarray analysis (Affymetrix Mouse Gene 1.0ST microarray). Cells treated with IFNγ were harvested every 60 min for a total of 12 h post-treatment for RNA isolation and microarray analysis (Affymetrix Mouse Gene 1.0ST microarray). In brief, the arrays were normalized using the gcRMA algorithm (Wu et al., 2004) and imported into Partek Genomics Suite (Partek, United States) for downstream analysis (Blanc et al., 2013). Temporal expression data for Nampt in MCMV-infected wild-type and *Tyk2*^-^*^/^*^-^ BMDM was generated using microarray. BMDM isolated from wild-type and *Tyk2*^-^*^/^*^-^ mice were cultivated for 7 days. Following 7 days, cells were infected with MCMV (MOI 1) and harvested 1–1.5 and 6–6.5 h post-infection followed by RNA isolation (RNeasy Midi kit, Qiagen) and microarray analysis (Affymetrix Mouse Gene 1.0ST microarray). Time course microarray analysis data are compliant with the National Centre for Biotechnology Information Gene Expression Omnibus (GEO) (Edgar et al., 2002) under SuperSeries accession number GSE42505 (SubSeries numbers GSE42503, GSE42504) (GEO^1^). Macrophage microarray data of MCMV-infected wild-type and *Tyk2*^-/-^ BMDM is accessible through GEO under accession number GSE126867.

### Statistical Analysis of Time-Course Data

Prior to statistical analysis, gene expression data [0–24 h post-mCMV infection or poly(I:C) treatment] was smoothened by taking the mean of every consecutive pair of points, i.e., mean of score at 0 and 2 h defined the score at 1 (hour) and mean of 2 and 4 h defined the score at 3 (hours), 4 and 6 h defined the score at 5 (hours), 6 and 8 h defining the score at 7 (hours), and 8 and 10 h defining the score at 9 (hours). Smoothing was not done beyond 10 h as the gap to the next point at 24 h was too large and, thus, excluded from analysis. This type of smoothing preserves the patterns in the data while removing some of the fine scale rapid changes. To investigate whether the gene expression changed over time, the mean smoothed data (or non-smoothened for the 12 h-dataset) for each gene expression was compared to a straight horizontal line. This was achieved by fitting the data to an appropriate polynomial in time *t*, i.e., either a linear model (y = α + βt), or a quadratic model (y = α + βt + γt^2^) or a cubic model (y = α + βt + γt^2^ + δt^3^). If the coefficients other than the intercept (α) were significant then the model and hence the gene expression, must vary with time. Which model was chosen depended on the model fit as given by the R^2^ value – the higher the better. The great majority of the fits were very strong –R^2^ > 0.9. To determine then if the model deviated significantly for the horizontal, the significance attached to each of the coefficients (β,γ,δ) of the time variables was investigated. If anyone of these was significant at the *p* < 0.05 level or if any two were significant at the 0.05 < *p* < 0.1 level then we considered the model and the gene expression to vary significantly with time. Refer to Supplementary Tables S5–S27 for the results of the statistical analysis.

## Data Availability

All datasets generated for this study are included in the manuscript and/or the Supplementary Files.

## Author Contributions

WD, KR, and PG conceived and designed the experiments. WD and KR performed the experiments, and WD, KR, and PG performed the data analysis. WJ performed the statistical analysis of the time-course microarray analysis. BS contributed to the *Tyk2*^-^*^/^*^-^ experiments. WD and PG wrote the manuscript.

## Conflict of Interest Statement

The authors declare that the research was conducted in the absence of any commercial or financial relationships that could be construed as a potential conflict of interest.

## Footnotes

1 http://www.ncbi.nlm.nih.gov/geo/

## Supplementary Material

FIGURE S1 Nampt expression in macrophages is Jak/Stat-dependent and induced by IFNγ stimulation. Normalized temporal *Nampt* expression in IFNγ-treated BMDM. The expression was measured hourly over the first 12 h of treatment using microarray, and the expression levels were fitted to a cubic polynomal on time and assessed for statitical significance (temporal change over 12 h). ^###^*p* < 0.001 were considered to be significant.

FIGURE S2 Knock-down efficiency of Nampt and Sirt6 in NIH-3T3 cells. **(A)** Quantification of relative Nampt mRNA expression in NIH-3T3 cells following using qRT-PCR following siRNA treatment (6.25, 12.5, and 25 nM siRNA) (*n* = 3). **(B)** Quantification of relative Sirt6 mRNA expression in NIH-3T3 cells following using qRT-PCR following siRNA treatment (6.25, 12.5, and 25 nM siRNA) (*n* = 3). One-way ANOVA with a Dunnett’s multiple comparisons test was used to determine statistical significance. Bars represent standard error of the mean (SEM). ^∗^*p* < 0.05, ^∗∗^*p* < 0.01, and ^∗∗∗^*p* < 0.001 were considered to be significant (ns, not significant).

TABLE S1 Putative TF binding in murine Nampt promoter region (-1 kb upstream of Nampt gene): prediction done with PROMO online based tool.

TABLE S2 Putative TF binding in murine Sirt6 promoter region (-1 kb upstream of Sirt6 gene): prediction done with PROMO online based tool.

TABLE S3 Putative TF binding in human NAMPT promoter region (-1 kb upstream of NAMPT gene): prediction done with PROMO online based tool.

TABLE S4 Putative TF binding in human SIRT6 promoter region (-1 kb upstream of SIRT6 gene): prediction done with PROMO online based tool.

TABLE S5 Coefficients^a^ for fit of Nampt expression over time in MCMV infected BMDM (0–10 h).

TABLE S6 Coefficients^a^ for fit of Sirt6 expression over time in MCMV infected BMDM (0–10 h).

TABLE S7 Coefficients^a^ for fit of Nampt expression over time in poly(I:C) treated BMDM (0–10 h).

TABLE S8 Coefficients^a^ for fit of Sirt6 expression over time in poly(I:C) treated BMDM (0–10 h).

TABLE S9 Coefficients^a^ for fit of Nampt expression over time in MCMV infected BMDM (0–12 h).

TABLE S10 Coefficients^a^ for fit of Myd88 expression over time in MCMV infected BMDM (0–10 h).

TABLE S11 Coefficients^a^ for fit of p50 (Nfkb1) expression over time in MCMV infected BMDM (0–10 h).

TABLE S12 Coefficients^a^ for fit of p65 (Rela) expression over time in MCMV infected BMDM (0–10 h).

TABLE S13 Coefficients^a^ for fit of Trif (Ticam1) expression over time in MCMV infected BMDM (0–10 h).

TABLE S14 Coefficients^a^ for fit of Rig-I (Ddx58) expression over time in MCMV infected BMDM (0–10 h).

TABLE S15 Coefficients^a^ for fit of Mda5 (Ifih1) expression over time in MCMV infected BMDM (0–10 h).

TABLE S16 Coefficients^a^ for fit of Ips-1 (Mavs) expression over time in MCMV infected BMDM (0–10 h).

TABLE S17 Coefficients^a^ for fit of Sting (Tmem173) expression over time in MCMV infected BMDM (0–10 h).

TABLE S18 Coefficients^a^ for fit of cGas (Md21d1) expression over time in MCMV infected BMDM (0–10 h).

TABLE S19 Coefficients^a^ for fit of Myd88 expression over time in poly(I:C) treated BMDM (0–10 h).

TABLE S20 Coefficients^a^ for fit of p50 (Nfkb1) expression over time in poly(I:C) treated BMDM (0–10 h).

TABLE S21 Coefficients^a^ for fit of p65 (Rela) expression over time in poly(I:C) treated BMDM (0–10 h).

TABLE S22 Coefficients^a^ for fit of Trif (Ticam1) expression over time in poly(I:C) treated BMDM (0–10 h).

TABLE S23 Coefficients^a^ for fit of Rig-I (Ddx58) expression over time in poly(I:C) treated BMDM (0–10 h).

TABLE S24 Coefficients^a^ for fit of Mda5 (Ifih1) expression over time in poly(I:C) treated BMDM (0–10 h).

TABLE S25 Coefficients^a^ for fit of Ips-1 (Mavs) expression over time in poly(I:C) treated BMDM (0–10 h).

TABLE S26 Coefficients^a^ for fit of Sting (Tmem173) expression over time in poly(I:C) treated BMDM (0–10 h).

TABLE S27 Coefficients^a^ for fit of cGas (Md21d1) expression over time in poly(I:C) treated BMDM (0–10 h).
