# Supplementary figures and images for: Metabolic Regulators Nampt and Sirt6 Serially Participate in the Macrophage Interferon Antiviral Cascade
Source: Front Microbiol. 2019 Mar 4;10:355. doi: 10.3389/fmicb.2019.00355 (PMC6409323; doi:10.3389/fmicb.2019.00355)

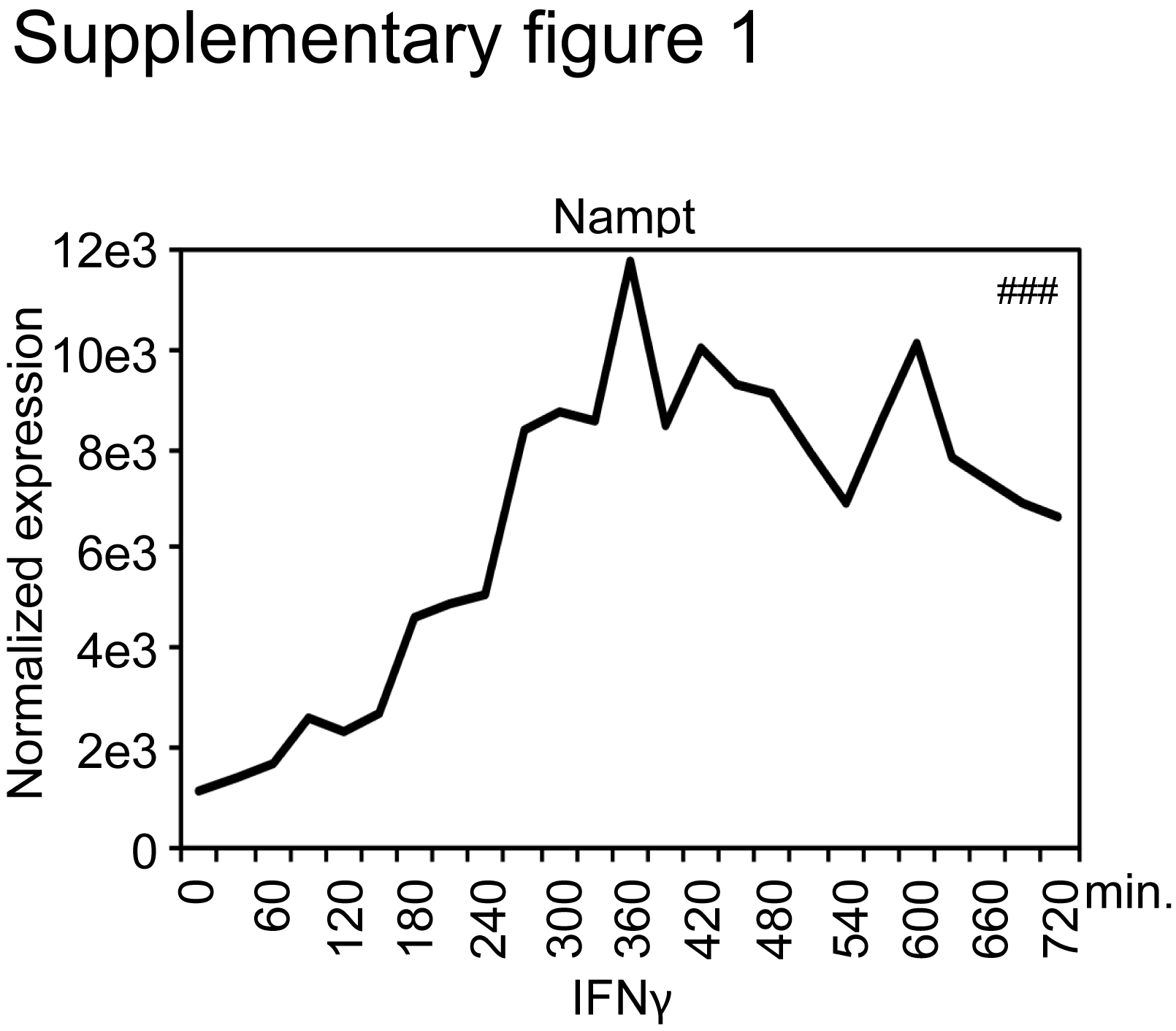

Supplement: FIGURE S1 — Nampt expression in macrophages is Jak/Stat-dependent and induced by IFNγ stimulation. Normalized temporal Nampt expression in IFNγ-treated BMDM. The expression was measured hourly over the first 12 h of treatment using microarray, and the expression levels were fitted to a cubic polynomal on time and assessed for statitical significance (temporal change over 12 h). ###p < 0.001 were considered to be significant. [file Image_1.TIF]

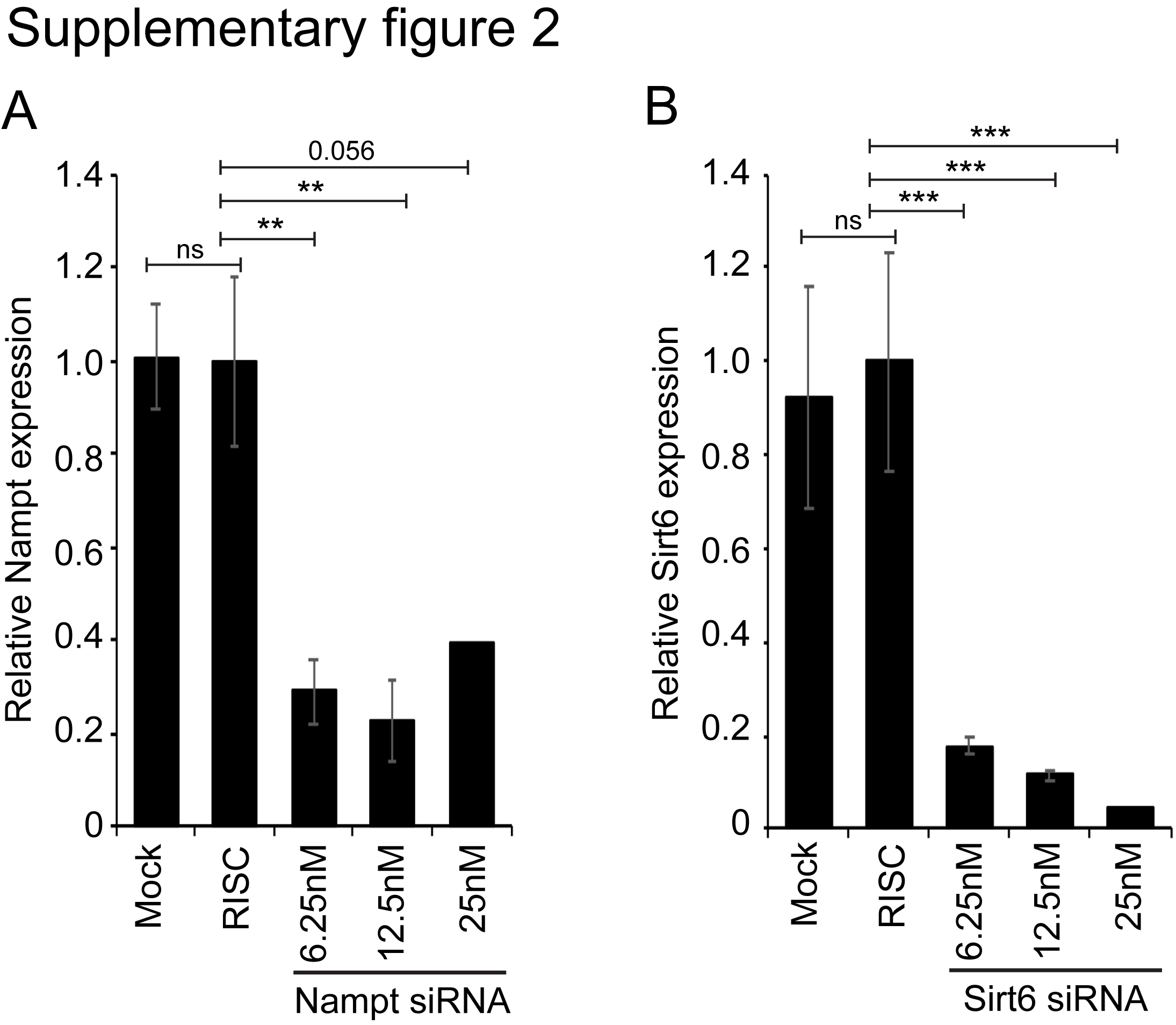

Supplement: FIGURE S2 — Knock-down efficiency of Nampt and Sirt6 in NIH-3T3 cells. (A) Quantification of relative Nampt mRNA expression in NIH-3T3 cells following using qRT-PCR following siRNA treatment (6.25, 12.5, and 25 nM siRNA) (n = 3). (B) Quantification of relative Sirt6 mRNA expression in NIH-3T3 cells following using qRT-PCR following siRNA treatment (6.25, 12.5, and 25 nM siRNA) (n = 3). One-way ANOVA with a Dunnett’s multiple comparisons test was used to determine statistical significance. Bars represent standard error of the mean (SEM). ∗p < 0.05, ∗∗p < 0.01, and ∗∗∗p < 0.001 were considered to be significant (ns, not significant). [file Image_2.TIF]
